# Superior gluteal artery as a reliable back door to embolize mycotic pseudoaneurysm of an isolated Internal Iliac artery

**DOI:** 10.1186/s42155-023-00367-w

**Published:** 2023-03-25

**Authors:** Hamad Aljutaili, Izzet Altun, Shahab A. Toursavadkohi, Nariman Nezami

**Affiliations:** 1 Division of Vascular and Interventional Radiology, Department of Diagnostic Radiology and Nuclear Medicine, University of Maryland School of Medicine, 22 S Greene St, N2W79A, Baltimore, MD 21201 USA; 2 Division of Vascular Surgery, Department of Surgery, University of Maryland School of Medicine, Baltimore, MD USA; 3 Experimental Therapeutics Program, University of Maryland Marlene and Stewart Greenebaum Comprehensive Cancer Center, Baltimore, MD USA

**Keywords:** Internal iliac artery, Superior gluteal artery, Pseudoaneurysm, Retrograde

## Abstract

**Background:**

Antegrade access through the origin of the internal iliac and direct percutaneous access under cross-sectional imaging guidance are commonly used for embolization of internal iliac artery aneurysms, pseudoaneurysms, or endoleaks. Here, we report superior gluteal artery retrograde access to treat internal iliac artery mycotic pseudoaneurysm in a patient with failed direct percutaneous access.

**Case presentation:**

We present a 65-year-old female with a history of diverticulitis and sigmoidectomy. Post-sigmoidectomy course was complicated by left common iliac artery (CIA) iatrogenic injury which required surgical ligation of the left CIA and graft placement. However, the graft was subsequently resection due to infection. Follow up CT imaging showed a 6 cm mycotic pseudoaneurysm (PSA) of the left internal iliac artery. Initially, the PSA sac was directly accessed and embolized under direct CT-guidance using Onyx. However, enlargement of the PSA sac was noted on one week follow-up CT images. Then, superior gluteal artery was accessed under ultrasound guidance, and the PSA sac and feeding vessels were re-embolized with coil and Onyx under fluoroscopy.

**Conclusion:**

Retrograde access through superior gluteal artery is a feasible and safe approach to embolize internal iliac aneurysms, pseudoaneurysms, or endoleaks, when the antegrade or direct percutaneous access is limited.

## Background

Iliac artery aneurysms (IAA) can affect 10%-20% of patients with aortic aneurysm (Brunkwall et al. 1989), whereas the prevalence of isolated internal Iliac artery (IIA) aneurysms is approximately 0.04% of all abdominal aortic aneurysm (Dix et al. 2005). IAA could be atherosclerotic or acquired which includes traumatic, mycotic, or post-surgical aneurysms. Forty percent of the patients with IIA are at the risk of rupture, associate with up to 60% mortality rate (Dix et al. 2005). Therefore, timely treatment of IIA aneurysm is critical.

Multiple approaches have been described to treat IIA aneurysm. Open repair, the traditional approach, is technically challenging, and associated with increased morbidity compared to endovascular technique (Chen et al. 2021) (Rana et al. 2014). Minimally invasive image guided methods include endovascular approach or direct percutaneous transgluteal puncture. Various endovascular techniques have been described for treatment of IIA aneurysms, including stenting across the ostium of the IIA distal embolization of the IIA with rustling proximal occlusion of the IIA, coil embolization of the aneurysm sac, or Amplatzer vascular plug(Chen et al. 2021). In anatomical suitable patients, the deployment of iliac branch device that permit the preservation of the flow of the hypogastric arteries and excludes the aneurysmal sac by blocking the outflow branches is a technique with promising results and low risk of pelvic ischemia.(Chen et al. 2021) However, routine antegrade endovascular approach might not be possible because of prior surgical ligation, jailed artery due to stenting, or very severe atherosclerotic disease.

Here, we report coil and Onyx embolization of a 6 cm iatrogenic mycotic pseudoaneurysm of left IIA by retrograde access through the superior gluteal artery (SGA) under ultrasound guidance, after failure of initial direct percutaneous access under computed tomography (CT) guidance.

## Case presentation

65-years-old female patient with history of diverticulosis developed large bowel obstruction and underwent sigmoidectomy which complicated by IIA injury required ilioiliac graft placement**.** Approximately 2 months later, the patient developed fever wand was hypotensive, Due to concern of graft infection, patient underwent exploratory laparotomy which confirmed graft infection, the graft was removed as well as abdominal washout was done. Following day left common iliac to left common femoral artery bypass was performed using left common femoral vein graft with closure of rectal stump leak. The patient developed acute bleeding from the graft area resulted with hemorrhagic shock, and controlled by graft ligation.

**Fig. 1:**
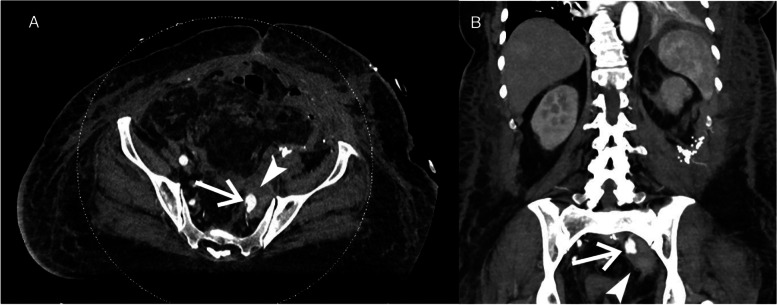
Initial CT angiogram of the left internal iliac artery mycotic pseudoaneurysm. **A**) Axial CT image and **B**) MPR coronal CT image shows left internal iliac artery mycotic pseudoaneurysm measuring 3 × 6 cm(arrow) and adjacent inflammatory changes (arrowhead)

**Fig. 2:**
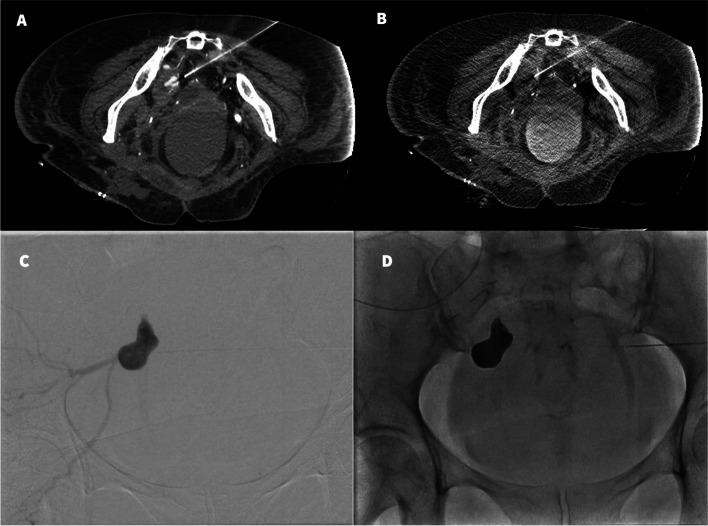
Direct percutaneous access to the mycotic pseudoaneurysm under CT guidance. **A**) Tip of the 20 G needle medial to the left internal iliac artery mycotic pseudoaneurysm sac on CT angiogram just before access to the sac. **B**) Direct access into the sac with 20 G needle. **C**) Contrast injection under fluoroscopy opacifies the left internal iliac artery mycotic pseudoaneurysm sac and feeding arteries. **D**) Onyx embolization of the mycotic pseudoaneurysm sac

**Fig. 3:**
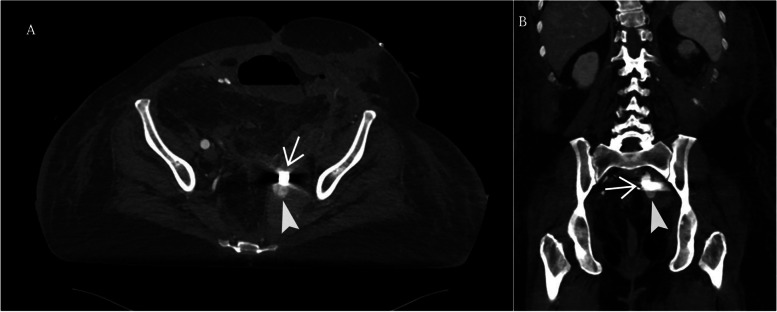
CT angiogram of the left internal iliac artery 7 days after embolization of mycotic pseudoaneurysm with onyx **A**) Axial and **B)** MPR coronal CT angiogram demonstrates enlarged left internal iliac artery mycotic pseudoaneurysm sac measuring 8 × 4 cm with partial filling of the sac (arrowhead) and prior onyx(arrow)

**Fig. 4:**
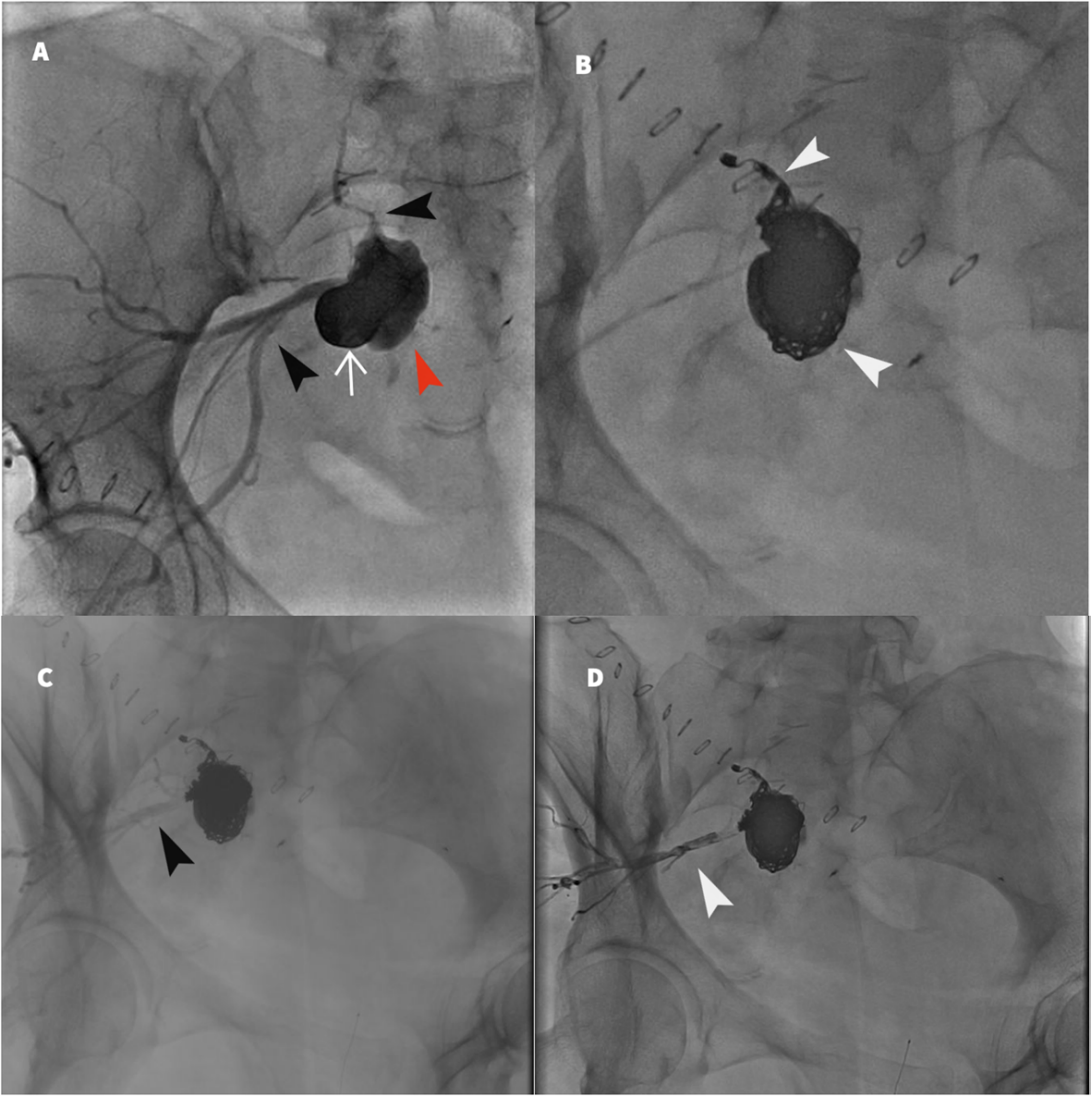
Pelvic angiogram through superior gluteal artery retrograde access of the left internal iliac artery. **A**) Initial angiogram demonstrates prior partial onyx embolization of the mycotic pseudoaneurysm (white arrow). Enlarging mycotic pseudoaneurysm sac (red arrowhead). Feeding vessels (black arrowhead). **B**) Coil embolization of the mycotic pseudoaneurysm sac and the inflow lateral sacral artery (white arrowheads). **C**) Pelvic angiogram post coil embolization demonstrates the outflow feeding vessels (black arrowhead). **D**) Onyx embolization of the outflow vessels (white arrowhead)

**Fig. 5:**
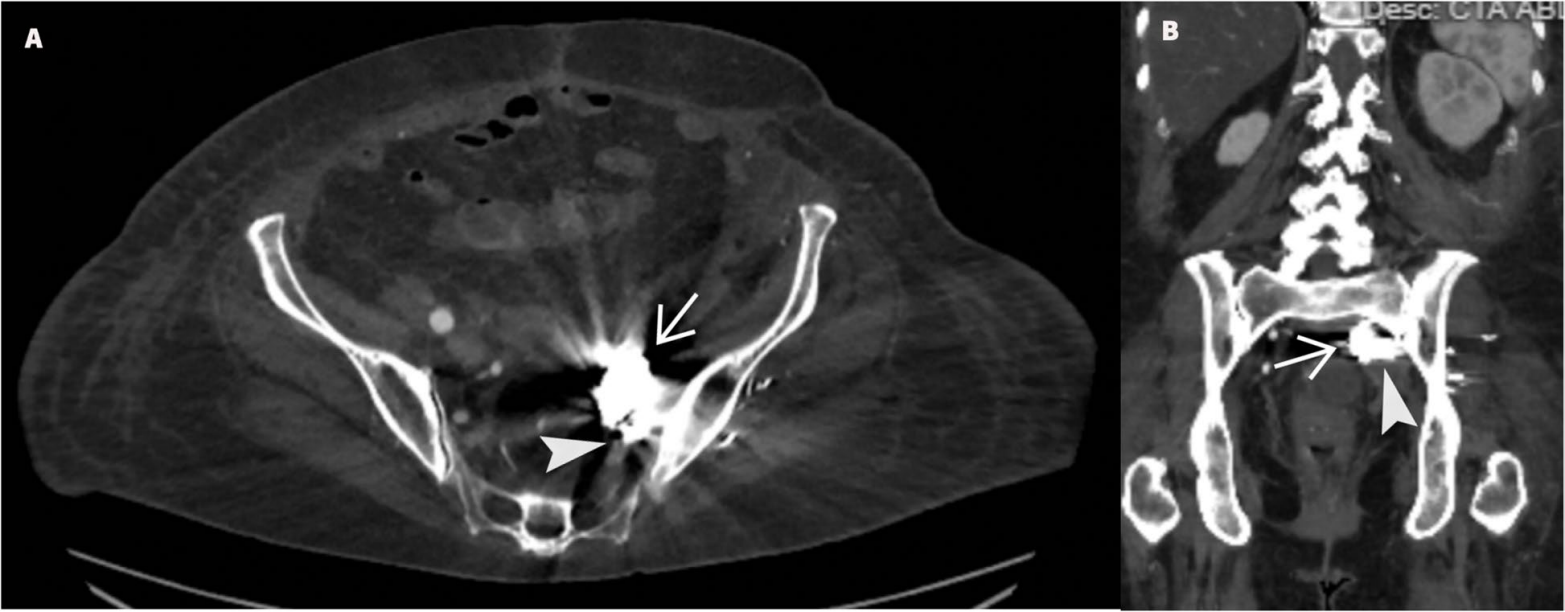
Follow up CT angiogram of the embolized left internal iliac artery mycotic pseudoaneurysm. **A**) Axial CT image and **B**) MPR coronal CT image of completely embolized left internal iliac artery mycotic pseudoaneurysm with onyx (arrow) coils (arrowhead) without contrast in the sac

Follow up CT scan showed a 3 × 6 cm mycotic pseudoaneurysm of the left IIA with adjacent inflammatory strandings (Fig. 1). This was initially embolized by CT guided direct access using 20 Gauge chiba needle (Cook, IN, USA) and onyx (Medtronic, MN, United states) (Fig. 2). Post embolization angiogram showed successful embolization of the sac (Fig. 2 D). However, the mycotic pseudoaneurysm continued to enlarge on follow-up CT angiogram and measured 8 × 4 cm with partial filling of sac (Fig. 3). The patient underwent second embolization of the mycotic pseudoaneurysm through SGA access. While patient was in prone position, the left SGA was accessed under color Doppler ultrasound guidance using a 21 Gauge 15 cm length needle (Cook, IN, USA). Contrast injection confirmed the left SGA position of the needle. A 0.018-inch Nitrex wire (Medtronic, MN, United states) was advanced into the left SGA. Subsequently, the needle was exchanged for a 5 French 15 cm transitional sheath. The dilator and microwire were traded for 2.8 Fr Progreat microcatheter (Terumo, NJ. United states). Then, the mycotic pseudoaneurysm was embolized with penumbra coils (Penumbra Inc. CA, Unites states) followed by onyx 18 embolization of the feeding vessels, iliolumbar artery and superior and inferior gluteal arteries (Fig. 4). The patient was also placed on an extended course of intravenous antibiotics. Follow-up CT Angiogram of the pelvis performed approximately 2 months after showed stable size and completely embolized left internal iliac mycotic pseudoaneurysm without any sign of enlargement and new contrast in the sac (Fig. 5).

## Discussion

We report successful retrograde access and embolization of the IIA mycotic pseudoaneurysm sac through SGA. In this case, direct percutaneous access puncture of the sac and onyx embolization failed to prevent pseudoaneurysm enlargement. This could be due to the nature of the mycotic pseudoaneurysm and the associated surrounding inflammatory changes. Retrograde access to the IIA through SGA provides a way to delineate all the inflow and outflow vessels as well as allow complete embolization of them. In our case, retrograde access to the IIA through the SGA ensured successful embolization of the IIA mycotic pseudoaneurysm sac and all the feeding vessels.

There have been few case reports of successful embolization of IIA aneurysm through retrograde SGA access for IIA aneurysm embolization including coil embolization of 7.3 cm left IIA (Ghasemi-Rad et al. 2022), embolization of 6.8 cm right and 4.8 cm left IIA aneurysms through bilateral SGA access in patient with aortobiiliac bypass with proximal hypogastric artery ligation(Kabutey et al. 2014), coil embolization of 7.5 cm partially thrombosed right IIA aneurysm in a jailed right IIA (Chi and Yan 2019) and embolization of ruptured IIA aneurysm secondary to type II Endo-leak (Patel et al. 2011).

The optimal treatment method for an IIA pseudoaneurysm is to exclude the sac, as well as the inflow and the outflow vessels to prevent pseudoaneurysm enlargement. This could be challenging to perform in cases where routine endovascular approach is impossible, therefore, there are two possible methods which both were used in our presenting case: direct percutaneous puncture of the sac under CT guidance and retrograde access using SGA as the backdoor.

Following embolization of IIA, buttock claudication that can range from 3%-40% in different studies (Pirvu et al. 2017), and sexual dysfunction which can be seen in 11%-38% of patients can occur(Pirvu et al. 2017). Other less common but devastating complications includes colonic ischemia, spinal cord ischemia/paralysis, gluteal compartment syndrome, perineal skin necrosis and bladder dysfunction (Rana et al. 2014) (Lin et al. 2009). In our case, no buttock claudication or other complications was reported by the patient.

## Conclusion

In conclusion, retrograde access to the internal iliac artery through SGA is a feasible and safe approach to embolize internal iliac artery mycotic pseudoaneurysm when anterograde endovascular approach or direct percutaneous access is not possible.

## Data Availability

Data sharing is not applicable to this article as no datasets were generated or analyzed during this case report.

## Abbreviations

IAA: I liac artery aneurysms
Internal Iliac artery: IIA
SGA: Superior gluteal artery
CT: Computed tomography

## References

[CR1] Brunkwall J, Hauksson H, Bengtsson H, Bergqvist D, Takolander R, Bergentz S-E (1989). Solitary Aneurysms of the Iliac Arterial System: An Estimate of Their Frequency of Occurrence. J Vasc Surg.

[CR2] Chen RJ, Vaes RHD, Qi SD, Westcott MJ, Robinson DR (2021). Modalities of Endovascular Management for Internal Iliac Artery Aneurysms. ANZ J Surg.

[CR3] Chi WK, Yan BP (2019). Direct Puncture of Superior Gluteal Artery Using a Doppler Ultrasound-Guided Needle to Access Jailed Internal Iliac Artery Aneurysm. J Vasc Surg Cases Innov Tech.

[CR4] Dix FP, Titi M, Al-Khaffaf H (2005). The Isolated Internal Iliac Artery Aneurysm—A Review. Eur J Vasc Endovasc Surg.

[CR5] Ghasemi-Rad M, Harshna VV, Christie ML, Zubin I (2022). Embolization of Large Internal Iliac Artery Pseudoaneurysm through a Retrograde Trans-Superior Gluteal Arterial Access. Tomography.

[CR6] Kabutey N-K, Siracuse JJ, Gill H, Kundi R, Meltzer AJ, Schneider DB (2014). Percutaneous Transgluteal Coil Embolization of Bilateral Internal Iliac Artery Aneurysms via Direct Superior Gluteal Artery Access. J Vasc Surg.

[CR7] Lin PH, Chen AY, Vij A (2009). Hypogastric Artery Preservation during Endovascular Aortic Aneurysm Repair: Is It Important?. Semin Vasc Surg.

[CR8] Patel SD, Perera A, Law N, Mandumula S (2011). A Novel Approach to the Management of a Ruptured Type II Endoleak Following Endovascular Repair of an Internal Iliac Artery Aneurysm. Br J Radiol.

[CR9] Pirvu A, Gallet N, Perou S, Thony F, Magne J-L (2017). Midterm Results of Internal Iliac Artery Aneurysm Embolization. Journal De Medecine Vasculaire.

[CR10] Rana MA, Kalra M, Oderich GS, de Grandis E, Gloviczki P, Duncan AA, Cha SS, Bower TC (2014). Outcomes of Open and Endovascular Repair for Ruptured and Nonruptured Internal Iliac Artery Aneurysms. J Vasc Surg.

